# Short-term effects of temperature on the abundance and diversity of magnetotactic cocci

**DOI:** 10.1002/mbo3.7

**Published:** 2012-03

**Authors:** Wei Lin, Yinzhao Wang, Yongxin Pan

**Affiliations:** 1 Biogeomagnetism Group, Paleomagnetism and Geochronology Laboratory, Key Laboratory of the Earth's Deep Interior, Institute of Geology and Geophysics, Chinese Academy of SciencesBeijing 100029, China; 2 France-China Bio-Mineralization and Nano-Structures Laboratory, Institute of Geology and Geophysics, Chinese Academy of SciencesBeijing 100029, China

**Keywords:** Abundance, diversity, magnetotactic bacteria, temperature, 16S rRNA genes

## Abstract

Temperature is one of the most important climate factors that can regulate the activity and growth of organisms. However, it is so far unclear how temperature influences the abundance and community composition of magnetotactic bacteria (MTB) that mineralize intracellular magnetite and/or greigite magnetosomes and play significant roles in the global iron cycling and sediment magnetization. To address this specific problem, in this study we have assessed the impact of temperature on freshwater magnetotactic cocci through laboratory microcosm simulations. Microcosms containing MTB were exposed to four constant temperatures ranging from 9°C to 37°C. After 10 days and 28 days of incubation, no significant differences in abundance were detected in microcosms at 9°C, 15°C, and 26°C (Student's *t*-test, *P* > 0.05); however, microcosms exposed to 37°C exhibited a significant decrease of magnetotactic cocci abundance (*P* < 0.05). Dendrogram analysis of community-amplified ribosomal DNA restriction analysis (community ARDRA) banding patterns distinguished the 37°C samples from samples at lower temperatures regardless of incubation periods. Furthermore, clone library analysis revealed that most of the operational taxonomic units (OTUs) detected in samples from 9°C to 26°C were absent from the 37°C microcosms, whereas six OTUs were exclusively detected in the 37°C samples. Community compositions from four incubation temperatures were further compared using statistical phylogenetic methods (UniFrac and LIBSHUFF), which revealed that the 37°C samples harbored phylogenetically distinct MTB communities compared to those found in 9°C, 15°C, and 26°C samples. Taken together, our results indicate that elevated temperature can influence the abundance and diversity of dominant members of magnetotactic cocci. This linkage further infers that the abundance and diversity of MTB (e.g., based on the fossil magnetosomes) may be useful in reconstruction of paleotemperature.

## Introduction

The response of species to modern and historical climate changes, such as global warming, has been of longstanding interest to researchers (Walther et al. 2002; Root et al. 2003). As one of the most important climate change factors, temperature can regulate the growth rates and activity of organisms, and thus may directly or indirectly impact on the structures of biological community (Matthew 1915; Walther et al. 2002; Root et al. 2003; Bradford et al. 2008). The shifts in flora and fauna associated with global warming have already been observed (Root et al. 2003). Recently, by using experimental simulations (such as microcosm experiments) and field studies, a growing number of researches have shown that the temperature change can also influence the abundance and diversity of microorganisms (e.g. Norris et al. 2002; Adams et al. 2010; Braker et al. 2010; Castro et al. 2010).

Magnetotactic bacteria (MTB) are a diverse group of microorganisms that ubiquitously distribute in aquatic environments from freshwater to marine ecosystems (Bazylinski and Frankel 2004; Faivre and Schüler 2008). Special interest attaches to MTB as their formation of magnetosomes that are intracellular membrane-enveloped iron minerals of magnetite (Fe_3_O_4_) and/or greigite (Fe_3_S_4_). It is widely accepted that, under the influence of the earth's magnetic field, magnetosomes facilitate the navigation of MTB to their preferred microenvironments in chemically stratified aquatic environments (Frankel et al. 1997; Bazylinski and Frankel 2004). MTB are able to accumulate up to 2–3% iron by dry weight (Heyen and Schüler 2003; Faivre and Schüler 2008), and in particular microhabitats MTB even account for a significant proportion of the microbial biomass (up to 30%) and are supposed to play dominant ecological roles in these sediment layers (Spring et al. 1993; Simmons et al. 2004; Simmons and Edwards 2006). Preliminary estimation has shown that MTB may contribute to about 1–10% of the flux of iron in chemically stratified marine environments (Faivre and Schüler 2008), therefore MTB have an important function in the aquatic iron cycle (Simmons and Edwards 2007).

16S rRNA gene-based community analysis has revealed a great deal about the diversity of MTB. MTB are phylogenetically affiliated within the *Alphaproteobacteria*, *Deltaproteobacteria*, *Gammaproteobacteria*, phylum *Nitrospirae*, and the candidate division OP3 (Amann et al. 2007; Kolinko et al. 2011; Lefèvre et al. 2011b; Lin et al. 2011b). In most freshwater environments, magnetotactic cocci in the *Alphaproteobacteria* are the dominant populations of MTB (e.g., Spring et al. 1992; Spring et al. 1994; Flies et al. 2005a; Lin et al. 2009; Lin and Pan 2009b). Recent studies have shown that the distribution and community structures of MTB are regulated by various environmental factors such as salinity, nitrate, or sulphur compounds (Martins et al. 2009; Lin and Pan 2010; Lin et al. 2011a; Postec et al. 2011). However, little information is available concerning the effect of temperature on MTB communities. The temperature sensitivity of MTB is a topic of considerable interest because knowledge of the linkage between MTB communities and environmental temperature may be a potential proxy for understanding the influence of global warming on the aquatic iron cycling.

One of the most intriguing properties of MTB is their ability to form magnetosome fossils that can be preserved in sediments. These fossils (known as fossil magnetosomes or magnetofossils), on one hand, are stable carriers of natural remanent magnetization (Kirschvink and Chang 1984; Petersen et al. 1986; Snowball 1994; Dearing et al. 2001; Pan et al. 2005; Kopp et al. 2009), and on the other hand, are potential archives of paleoenvironmental information (Chang and Kirschvink 1989; Mandernack et al. 1999; Pan et al. 2004). So far the robust identification of the oldest magnetofossils traces back to the Cretaceous (Kopp and Kirschvink 2008). Several studies have shown evidences for linkages between magnetofossils and paleoenvironmental conditions, such as pore water oxygen level, organic carbon flux, and redox condition (Hesse 1994; Lean and McCave 1998; Yamazaki and Kawahata 1998; Kopp et al. 2007). However, the role of paleotemperature in determining the abundance and distribution of magnetofossils has not been addressed. Clearly, investigation of the temperature effect on present-day MTB populations will lead to reliable reconstructions of past environmental change based on magnetofossil records.

To address how temperature influences the abundance and diversity of MTB, in this study we compared the magnetotactic cocci community in microcosms from Lake Miyun near Beijing (site MY11), which were incubated at four constant temperatures (9°C, 15°C, 26°C, and 37°C) in the laboratory. Previous study has shown that large amount of MTB existed in site MY11 that belonged to magnetotactic cocci within the *Alphaproteobacteria* (Lin and Pan 2010). 16S rRNA gene-based fingerprinting technique, molecular phylogenetic approach, and statistical phylogenetic methods were applied to investigate the response of magnetotactic cocci to different environmental temperatures.

## Results

**Figure 1:**
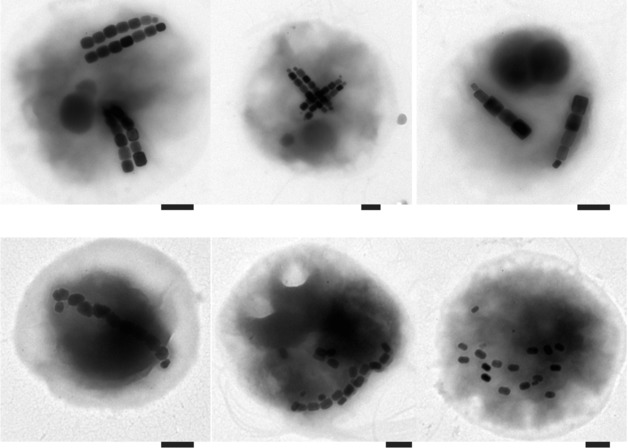
Representative transmission electron micrographs of various magnetotactic cocci from Lake Miyun near Beijing, China (Bars = 200 nm).

**Figure 2:**
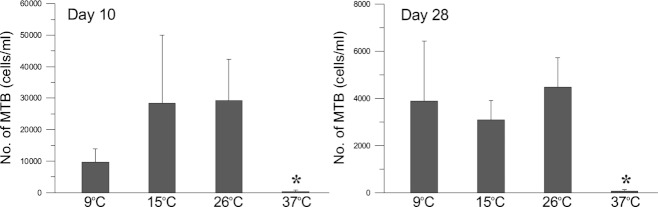
Abundances of MTB for 10-day and 28-day samples incubated at 9°C, 15°C, 26°C, and 37°C. Each data represent the average measurement of three replicate microcosms with error bars indicating the standard deviation. (*) indicates a significant (Student's *t*- test, *P* < 0.05) temperature effect on magnetotactic cocci abundance.

Transmission electron microscopy (TEM) observation revealed that magnetotactic cocci were the dominant MTB in all microcosms at four different temperatures (Fig. 1). The mean abundances of magnetotactic cocci ranged from 2.92 × 10^4^ cells mL^−1^ (26°C) to 0.03 × 10^4^ cells mL^−1^ (37°C) after 10 days of incubation, and from 0.45 × 10^4^ cells mL^−1^ (26°C) to 0.007 × 10^4^ cells mL^−1^ (37°C) after 28 days of incubation (Fig. 2). The lowest number was always found in the sediments incubated at 37°C, which were significantly lower than that under 9°C, 15°C, and 26°C (Fig. 2, Student's *t*-test, *P* < 0.05).

**Figure 3:**
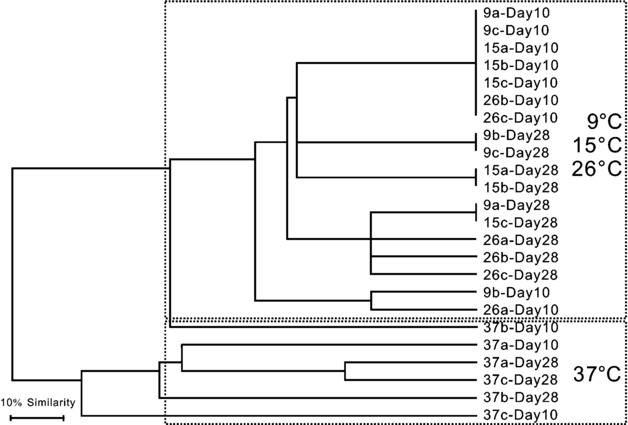
Unweighted-pair group method using average linkages (UPGMA) cluster analysis of community ARDRA banding patterns for 9°C, 15°C, 26°C, and 37°C samples incubated after 10 days and 28 days (a, b, and c refer to three replicate microcosms incubated at same temperature).

Community-amplified ribosomal DNA restriction analysis (Community ARDRA) was performed to provide an overview of the community structure from different incubation time and temperatures. ARDRA banding patterns were used to construct dendrogram, which revealed that, in general, magnetotactic cocci communities were conserved through replicate microcosms, time periods, and temperatures at 9°C, 15°C, and 26°C (Fig. 3). In contrast, communities from 37°C samples, except one 10-day sample (sample 37b-Day 10), were dramatically changed at both 10 days and 28 days of incubation and formed a separate cluster (Fig. 3).

**Figure 4:**
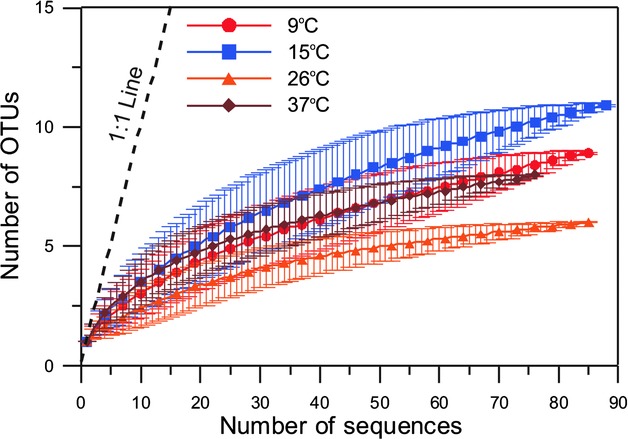
Rarefaction curves at 98% sequence similarity level for 9°C, 15°C, 26°C, and 37°C samples after 10 days of incubation. Error bars indicate 95% confidence intervals. For clarity, every third sample was plotted.

Since the community ARDRA analysis revealed similar banding patterns for samples incubated for 10 days and 28 days, we selected the 10-day samples for a more detailed community analysis using 16S rRNA gene clone libraries. Libraries were constructed and a total of 338 quality sequences (average length of 837 bp) were obtained from 12 samples. All of them were most similar to known magnetotactic cocci within the *Alphaproteobacteria* (≥91% similarity, Table 1). These sequences were grouped into operational taxonomic units (OTUs) based on their genetic distance using the furthest neighbor method and a similarity threshold of 98%. Consequently, the 338 sequences were assigned to 21 different OTUs. Figure 4 showed the rarefaction curves created for each incubation temperatures. A saturation, which indicated more complete coverage in sampled biodiversity, was approached for all four temperatures, suggesting that we have sampled most magnetotactic cocci taxa at 98% similarity scale.

**Table 1:** Nearest GenBank relatives for each OTUs received in this study

OTU	Length (bp)	Nearest GenBank relative (accession no. of relative)	Location	% Identity	Reference
OTU-G11	837	Uncultured magnetococcus sp. clone OTU8 (GQ468509)	Lake Miyun, Beijing, China	97	Lin and Pan (2010)
OTU-J24	837	Uncultured magnetococcus sp. clone OTU8 (GQ468509)	Lake Miyun, Beijing, China	97	Lin and Pan (2010)
OTU-H15	837	Magneticcoccus (CS308, X61607)	Lake Chiemsee, Munich, Germany	97	Spring et al. (1992)
OTU-E3	837	Uncultured magnetococcus sp. clone 17 (EU780677)	Lake Miyun, Beijing, China	96	Lin et al. (2009)
OTU-F20	837	Uncultured magnetococcus sp. clone 17 (EU780677)	Lake Miyun, Beijing, China	98	Lin et al. (2009)
OTU-E15	837	Uncultured magnetococcus sp. clone 17 (EU780677)	Lake Miyun, Beijing, China	96	Lin et al. (2009)
OTU-C9	837	Magnetic coccus (CS103, X61605)	Lake Chiemsee, Munich, Germany	96	Spring et al. (1992)
OTU-E26	837	Uncultured magnetococcus sp. clone OTU29 (GQ468510)	Lake Miyun, Beijing, China	98	Lin and Pan (2010)
OTU-A10	837	Uncultured magnetococcus sp. clone 17 (EU780677)	Lake Miyun, Beijing, China	95	Lin et al. (2009)
OTU-H2	838	Magnetic coccus (CS103, X61605)	Lake Chiemsee, Munich, Germany	96	Spring et al. (1992)
OTU-F30	838	Uncultured magnetococcus sp. clone 17 (EU780677)	Lake Miyun, Beijing, China	95	Lin et al. (2009)
OTU-E29	837	Uncultured magnetococcus sp. clone 17 (EU780677)	Lake Miyun, Beijing, China	94	Lin et al. (2009)
OTU-B20	838	Uncultured magnetococcus sp. clone Y2 (GQ338464)	Lake Miyun, Beijing, China	95	Lin and Pan (2009a)
OTU-J22	837	Uncultured magnetococcus sp. clone XSE-42 (EF379385)	Huiquan bay, Shandong, China	95	Pan et al. (2008)
OTU-C8	837	Uncultured magnetococcus sp. clone OTU13 (GQ468512)	Lake Miyun, Beijing, China	95	Lin et al. (2009)
OTU-L18	837	Magnetic coccus 16S rRNA gene (CS81, X81184)	Lake Chiemsee, Munich, Germany	98	Spring et al. (1994)
OTU-D6	836	Uncultured magnetococcus sp. clone F1 (GQ338449)	Lake Miyun, Beijing, China	91	Lin and Pan (2009a)
OTU-J21	837	Uncultured magnetococcus sp. clone OTU17 (GQ468515)	Lake Miyun, Beijing, China	98	Lin et al. (2009)
OTU-L30	836	Uncultured magnetococcus sp. clone M-67 (EF371491)	Jiaozhou bay, Shandong, China	92	Xing et al. (2008)
OTU-L22	836	Magnetic bacterium small subunit rRNA gene, strain rj58 (Y13211)	Itaipu Lagoon, Rio de Janeiro state, Brazil	94	Spring et al. (1998)
OTU-G15	836	Magnetic bacterium small subunit rRNA gene, strain rj12 (Y13215)	Itaipu Lagoon, Rio de Janeiro state, Brazil	96	Spring et al. (1998)

**Figure 5:**
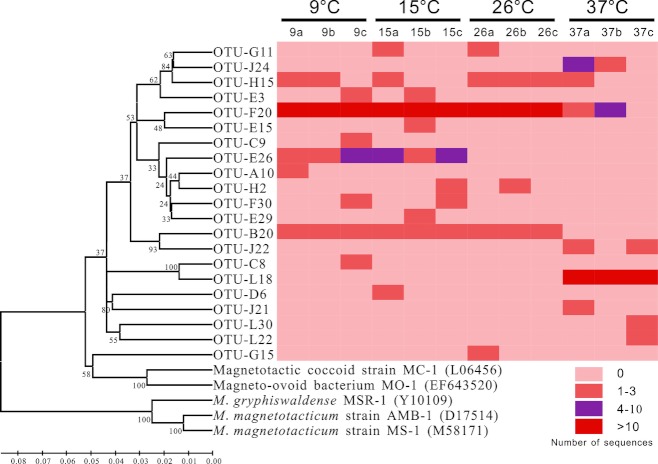
Phylogenetic tree and heatmap displaying the abundance of OTUs with ≥98% similarity in 12 microcosms after 10 days of incubation. The left is a neighbor-joining tree composed of representative sequences from each of 21 OTUs identified in this study, which is linearized assuming equal evolutionary rates in all lineages. Abundance of OTUs in each microcosm is indicated by different colors in the plot to the right.

Figure 5 illustrated the phylogenetic architecture of the 21 OTUs and their abundance in each of 12 microcosms. The two most abundant OTUs were OTU-F20 and OTU-L18 that accounted for 76.6% of all sequences. OTU-F20 was abundant in samples incubated at 9°C (75.6%), 15°C (69.7%), and 26°C (83.7%). This OTU has been found to be abundant in our previous studies in the same location (Table 1). In the case of 37°C samples, OTU-L18 was the dominant OTU representing 66.2% of identified sequences (Fig. 5). Although 98% similar to one sequence (X81184) found in Germany (Spring et al. 1994), OTU-L18 was phylogenetically distinct (<92% similarity) from all MTB sequences previously derived from Lake Miyun (Table 1). Of total 21 OTUs, 10 OTUs were only found at one of the 12 microcosms, and no OTUs were shared across all 12 microcosms (Fig. 5). The distribution of sequences was relatively similar among samples incubated at 9°C, 15°C, and 26°C; however, a high degree of variability in the taxonomic structure of sequences from 37°C was detected (Fig. 5).

**Figure 6:**
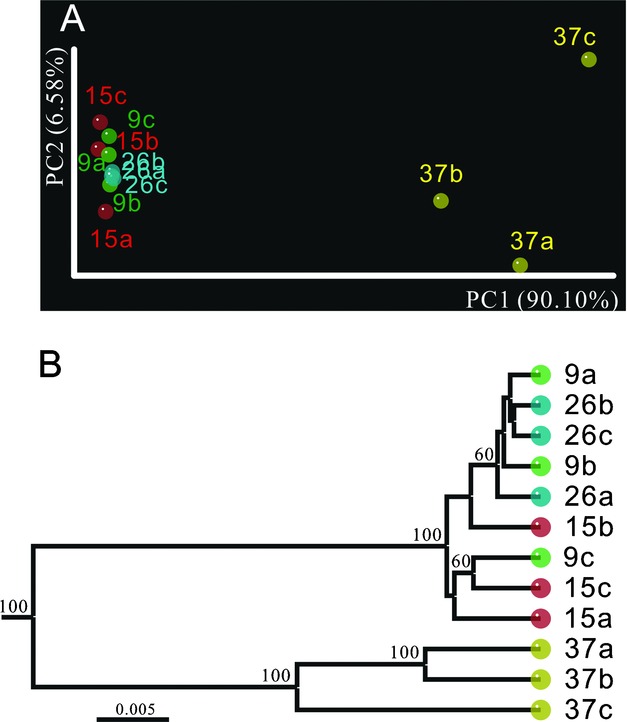
Effect of temperature on the communities of magnetotactic cocci from each 10-day microcosm. PCoA (A) and Jackknife environment clusters (B) based on the matrix of pairwise distances between communities generated using the weighted UniFrac. The values at each node in (B) indicate the percent Jackknife support based on 1000 random sampling, values of greater than 50% are shown. The bar in (B) represents a weighted UniFrac distance of 0.005.

The relationships between the 12 libraries were further statistically compared using the weighted UniFrac program to test the influence of temperatures on the phylogenetic composition of magnetotactic cocci. To more effectively visualize this influence, principal coordinates analysis (PCoA) was performed to examine the phylogenetic similarity between all samples (Fig. 6). In accordance with community ARDRA results, PCoA analyses clearly revealed that the 37°C samples harbored phylogenetically distinct MTB communities compared to those found in 9°C, 15°C, and 26°C samples, where PCoA axis 1 explained 90.1% of the variation (Fig. 6A). In addition, there was a very high level of similarity in the phylogenetic structure across samples incubated from 9°C to 26°C. These results were further corroborated by the Jackknife sample clusters analysis that exhibited the highest level of Jackknife support for the branch between 37°C samples and other samples (100% Jackknife value, Fig. 6B). LIBSHUFF program was used to test the significance of the differences between the samples incubated under different temperatures (Table 2). Using a corrected *P*-value of 0.0041 for multiple comparisons (0.05/12 pairwise comparisons), 37°C samples significantly differed from the other samples (*P* < 0.0001), while MTB communities between 9°C, 15°C, and 26°C were not significantly different (*P* ≥ 0.0041 in all cases, Table 2). Taken together, these results indicated that elevated temperature (37°C) can cause a major shift in community structure of dominant members of MTB.

**Table 2:** *P*- values estimating similarity among each treatment generated using LIBSHUFF

	*P*- value comparison of heterologous library (*Y*) with *X*^a^			
	
Community (*X*)	9°C	15°C	26°C	37°C
9°C	/	0.9056	0.0076	<0.0001*
15°C	0.6215	/	0.0041	<0.0001*
26°C	0.8636	0.9631	/	<0.0001*
37°C	<0.0001*	<0.0001*	<0.0001*	/

a P-values comparing either *X* to *Y* or *Y* to *X* indicate that the two communities are significantly different (*P* < 0.0041, 0.05/12 pairwise comparisons).

* Significantly different.

## Discussion

Understanding the linkage between the abundance and diversity of MTB and their living environmental temperature is of great interest. However, assessment of this linkage through direct field studies is somewhat difficult because of high variability of environmental factors. Microcosm studies can offer good opportunities to examine the impact of major factors on microorganisms, in which the experimental conditions can be well controlled. In the present study, we used microcosm systems to investigate possible influence of temperature on the MTB community. During the experiments, each treatment started with the same well-homogenized initial sediment samples, and the temperature is the only variable within each treatment.

The abundance of magnetotactic cocci, as estimated by direct counts using hanging-drop approach, was highly variable, resulting in high standard deviations in samples at each temperature (Fig. 2). This is a widely recognized limitation of this method. However, the advantage of this method is the differentiation between active and dead magnetotactic cells and thus effectively reflects the short-term dynamics of community influenced by environmental changes. Therefore, the hanging-drop approach is a useful tool for enumeration of live MTB and is widely applied in various magnetotactic bacterial researches (e.g., Flies et al. 2005b; Jogler et al. 2010; Lefèvre et al. 2011a). The abundances of magnetotactic cocci found in the present study fall within the range of values reported previously (Lin et al. 2008; Lin et al. 2009). The striking finding in this study is that 37°C appears a critical temperature for the abundance of magnetotactic cocci. After 10-day and 28-day incubations, samples at 37°C consistently harbor the lowest number of MTB, and this decrease is significant compared to microcosms at other temperatures. This result suggests that MTB communities in Lake Miyun do not tolerate elevated temperature (e.g., ≥37°C) and the warming stress (from 26°C to 37°C) will significantly reduce the abundance of magnetotactic cocci.

Temperature-dependent changes in the diversity of magnetotactic cocci were observed in the phylogenetic affiliations of OTUs. The fact that sequences retrieved in this study were amplified from magnetic enrichments using magnetotactic cocci specific primers indicated that all these sequences were from magnetotactic cocci, which was further confirmed by the high similarity of the retrieved sequences to known MTB as shown in the Table 1. Overall, MTB community structure changed significantly at the high temperature (37°C) but not at the low temperatures (9–26°C). For example, several OTUs, such as OTU-F20 and OTU-B20, which were consistently dominant from 9°C to 26°C, were decreased or absent at 37°C. In contrast, OTU-L18 and OTU-J24, absent at the lower temperatures, became dominant at 37°C (Fig. 5). This result was confirmed by using phylogeny- and distance-based comparison tools (UniFrac and LIBSHUFF), which clearly revealed that 37°C communities were significantly different from MTB at lower temperatures. Since the water temperature of Lake Miyun normally ranges between 8°C and 28°C from spring to fall (Li 2009), the high community similarity among 9°C, 15°C, and 26°C samples suggests that the dominant members of MTB appear to be well adapted to ambient temperatures in their ecosystems.

The community fluctuation of *Alphaproteobacteria* magnetotactic cocci observed here is similar to the changes of soil microbial communities, as revealed by Bárcenas-Moreno et al. (2009), which were more prominent above 35°C but were less evident below 30°C. Our results suggest that there is a selection pressure for magnetotactic cocci under high temperature, that is, the community dynamics of MTB at elevated temperature may be due to exceeding physiological thresholds of many populations, thus selecting for a group of tolerant species. Another reasonable explanation is that, across the temperature gradient, different magnetotactic cocci populations may have distinct competitive powers for available resources and the warm-tolerant OTUs (such as OTU-L18) have a strong competitive advantage to rapidly outcompete the original community at elevated temperature (Hall et al. 2008). Alternatively, at elevated temperature, the thermally adapted populations can metabolize the substrates that cannot be utilized by members of MTB at lower temperatures (Zogg et al. 1997).

It should be noted that a few specific taxa of magnetotactic cocci might not be detected through the specific primers used here (Lin and Pan 2009a). In spite of this inherent limitation, in this study, we totally detected 21 different OTUs of cocci, representing the highest level of MTB diversity in Lake Miyun sediments to date. In addition, the phylogenetic affiliations of retrieved OTUs are comparable with previous results that are acquired by bacterial universal primers (Table 1) (Lin et al. 2008; Lin et al. 2009; Lin and Pan 2010). Therefore, our results confirm the efficiency of these primers that can amplify a broad spectrum of freshwater *Alphaproteobacteria* magnetotactic cocci.

Simmons et al. (2007) have revealed a seasonal shift of MTB communities in a brackish seasonally stratified pond. The present study has shown that a high temperature (37°C) has short term and significant effects on both abundance and diversity of dominant MTB populations in Lake Miyun. However, since the current study focused on magnetotactic cocci from Lake Miyun, we can not exclude the possibility that MTB communities from other environments may exhibit distinct temperature sensitivities, like those MTB detected from hot springs (Lefèvre et al. 2010). Therefore, further more comprehensive studies are needed to fully understand the relationship between environmental temperature, as well as other temperature-dependent factors, and MTB communities from various habitats.

In summary, microcosm-based analyses have shown that the abundance and diversity of magnetotactic cocci from Lake Miyun changed significantly at elevated temperature (37°C) but not at the low temperatures (9–26°C). These results suggest that we may link the density and diversity of MTB to environmental temperature. These linkages may help us to better understand the dynamics of MTB communities in response to the climate change, and perhaps further provide useful clues for the paleoenvironmental temperature reconstruction based on the fossil magnetosomes preserved in sediments.

## Materials and Methods

### Site description and sediment sampling

Surface sediment samples were collected from the freshwater Lake Miyun near Beijing, China. The site MY11 was the same as that used for previous study (Lin and Pan 2010). Habitat conditions of Lake Miyun were reported previously (Pan et al. 2009; Lin et al. 2011a). Briefly, the water depth of sampling station was approximately 2–4 m. The mean annual temperature of Lake Miyun is ca. 10.5°C, with the highest temperature around 38°C (Liu et al. 2001). The temperature and pH of surface sediment during the sampling time were 22°C and 7.4, respectively. Additional physical–chemical characteristics of samples from site MY11 have been reported elsewhere (Lin and Pan 2010).

### Establishment of microcosm

In the present study, a microcosm experiment was designed with four different temperatures (9°C, 15°C, 26°C, and 37°C; range ±1°C). Microcosms were prepared by adding 300 mL of thoroughly homogenized sediment to 600-mL plastic bottles covered with 200 mL of lake water in the laboratory. Microcosms were then randomly divided into four treatments under different temperatures for up to 28 days. For each treatment, three replicated microcosms were used, resulting in a total of 12 microcosms. All microcosms were covered with aluminum foil to prevent any exposure to light. In order to maintain the water content, water loss through evaporation was replenished every 2 or 3 days using filter-sterile lake water. Therefore, during the experiments, each treatment started with the same well-homogenized initial sediment samples.

### Measurement of abundance of magnetotactic cocci

After 10 days and 28 days of incubation, the number of magnetotactic cocci was directly counted via hanging-drop method under light microscope using a protocol described previously (Flies et al. 2005b; Jogler et al. 2010). For each microcosm, three replicate drops were counted to calculate the average number of magnetotactic cocci.

### Magnetic enrichment of MTB and TEM observation

In order to investigate the effect of different temperatures on the diversity of magnetotactic cocci, live MTB cells were magnetically enriched through capillary racetrack method after 10 days and 28 days of incubation, respectively (Wolfe et al. 1987). Approximate 2 mL of sediment that contained MTB was placed in the reservoir of each capillary tube. For each microcosm, 5–10 capillaries were used. After 2 h of incubation under magnetic field, the tip of capillary was snapped off and the enriched cells were removed with a sterile syringe. For TEM observation, 20 μl of MTB enrichments were deposited on Formvar-carbon-coated copper grids. After waiting for 1 h, the remaining solution was wicked away using a piece of filter paper. The samples were then rinsed with sterile distilled water. Specimens were imaged using a JEM-1400 microscope operating at 80 kV (JEOL Corporation, Japan). The rest enrichments were frozen at −20°C prior to molecular analysis.

### PCR amplification and community ARDRA analyses

Since the dominant MTB from site MY11 were affiliated within *Alphaproteobacteria* magnetotactic cocci according to our previous study (Lin and Pan 2010), here the 16S rRNA gene sequences of MTB were amplified using specific primers (FMTCf and FMTCr) following a protocol described previously (Lin and Pan 2009a) with minor modifications. Each amplification was performed in volume of 40 μl containing 20 μl of DreamTaq PCR Master Mix (MBI Fermentas), 1.6 μl of 10 μM of each primer, 5 μl of template MTB enrichment, and 11.8 μl of nuclease-free water. The reaction was carried out under the following conditions: 5 min of initial denaturation at 95°C followed by 40 cycles of 30 sec at 94°C, 30 sec at 45°C, and 1 min at 72°C, with a final extension at 72°C for 10 min. Negative controls were used in all PCRs. Triplicate reactions were pooled for each sample before electrophoresis performed in 0.8% agarose in Tris-acetate EDTA buffer and PCR products were then extracted from the agarose gel by using a gel purification kit (Omega Bio-Tek, Inc, Lilburn, GA). Community ARDRA (Tiedje et al. 1999) was used here to provide an overview of the microbial community structures under different collection time and temperatures. Purified PCR products were digested with restriction endonucleases *Msp*I plus *Rsa*I at 37°C overnight (Lin et al. 2009). Community ARDRA patterns were photographed using gel documentation system INFINITY (Vilber Lourmat, Marne-la-Vallée, France) after electrophoresis for 40 min at 110 V in 3% agarose gels. Gels were analyzed through Bio-1D version 12.08 software package (Vilber Lourmat, Marne-la-Vallée, France) and the ARDRA similarity matrix was generated using Jaccard similarity coefficient with 3.00 percentage of tolerance. Clustering of the ARDRA similarity matrix was performed using the unweighted-pair group method using average linkages (UPGMA) cluster analysis.

### DNA cloning, sequencing, and sequences analysis

For a more comprehensive phylogenetic characterization between different temperatures, clone library analysis was performed for the 10-day samples. Purified PCR products of MTB were ligated with the pMD19-T vector (TaKaRa, Japan) and cloned into the chemically DH5α competent cells (Tiangen, China) according to the manufactures’ instructions. Thirty clones were randomly picked up per library and were subjected to sequencing. The 12 clone libraries yielded a total of 360 sequences. Vector contamination and low-quality sequences were removed. Sequences were then screened for chimeras using the Greengenes chimera-check tool (Bellerophon server) (Huber et al. 2004; DeSantis et al. 2006b). In addition, 15 sequences that contained unalignable inserts were removed. The remaining 338 sequences were aligned and manually edited through NAST aligner (DeSantis et al. 2006a). Distance matrices were calculated by using the DNADIST program within the PHYLogeny Inference Package (PHYLIP) (Felsenstein 1989), which was used as the input file for mothur v.1.15.0 (Schloss et al. 2009). The number of OTUs was calculated on the basis of 98% similarity using the furthest neighbor algorithm of the mothur v.1.15.0. Rarefaction analysis was performed using the program aRarefactWin available at http://www.uga.edu/strata/software/index.html.

The phylogenetic distances between MTB communities from each microcosm were determined using the weighted Fast UniFrac algorithm, a quantitative measurement based on the fraction of branch length unique to any community to the total branch length in a phylogenetic tree (Hamady et al. 2010). A neighbor-joining phylogenetic tree generated with MEGA v4.0 (Tamura et al. 2007) rooted with the 16S rRNA gene sequence of “*Candidatus* Magnetobacterium bavaricum” was used as the input file. PCoA and Jackknife environment clusters were performed to classify MTB communities. In order to identify whether the MTB communities were changed across different temperatures, replicate libraries were pooled for each temperature and the sequences were then compared using the LIBSHUFF program in the mothur v.1.15.0 according to Good's homologous and heterologous coverage (Schloss et al. 2004; Schloss et al. 2009). The LIBSHUFF program can quantitatively compare two libraries to determine if they differ significantly from each other.

### Accession numbers

The sequence data have been submitted to the DDBJ/EMBL/GenBank databases under accession number JN188474–JN188811.
